# WHO’s end of TB targets: unachievable by 2035 without addressing under nutrition, forced displacement, and homelessness: trend analysis from 2015 to 2022

**DOI:** 10.1186/s12889-024-18400-5

**Published:** 2024-04-04

**Authors:** Birhanu Ayenew, Dawit Misganaw Belay, Yegoraw Gashaw, Wondimu Gimja, Yimenu Gardie

**Affiliations:** 1 Department of Adult Health Nursing, College of Health Science, Assosa University, Assosa, Ethiopia; 2 Department of Midwifery, College of Health Sciences, Assosa University, Assosa, Ethiopia; 3 Department of Pediatric and Child Health Nursing, College of Health Science, Assosa University, Assosa, Ethiopia

**Keywords:** End TB, WHO End TB target, Undernutrition, Forced displacement, Homelessness

## Abstract

Tuberculosis (TB) remains a significant global health challenge, despite the World Health Organization (WHO) actively working towards its eradication through various initiatives and programs. Undernutrition, forced displacement, and homelessness worsen TB’s burden and challenge control efforts; however, there is still no adequate research that shows the trend of these underlying factors to attain the WHO’s ambitious TB targets. So, this study aims to analyze the trend analysis of these underlying factors worldwide from 2015 to 2022 and their impact on the feasibility and implications of reaching the End TB targets by 2035. We utilized international databases, including UNHCR, FAO, and WHO reports, as secondary data sources. Data were extracted chronologically from 2015 to 2022 to illustrate trends in undernutrition, forced displacement, and homelessness on a global level.

This trend analysis reveals that undernutrition, forced displacement, and homelessness have worsened over time. Undernutrition rose from 8.4 to 9.8% globally between 2015 and 2021, affecting 22.7 million additional individuals each year. In 2022, undernutrition affected 735 million people globally. Africa (21.9%) and Asia (10.6%) had the highest rates, while Western Europe and North America had lower rates than the global average: 3.4% and 2.5%, respectively.

Similarly, the global rate of forced displacement increased from 65.1 million people in 2015 to 108.4 million in 2022, a 21% increase from 2021. This means that an extra 19 million people were displaced in 2021. Globally, homelessness, affecting 1.6 billion people, has worsened over time. Despite being a highly vulnerable group to TB, homeless individuals are often neglected in TB control efforts. Our findings underscore the critical importance of addressing undernutrition, forced displacement, and homelessness in achieving the World Health Organization’s ambitious End TB targets by 2035, as highlighted through trend analysis from 2015 to 2022. Implementing policies focusing on nutrition, stable housing, and the challenges faced by displaced populations is imperative for progress toward a TB-free world.

## Background

Tuberculosis (TB) stands as a significant global health threat, causing a substantial burden of disease and mortality. It ranks among the top 10 deadliest infectious diseases worldwide [1, 2]. According to the World Health Organization (WHO), there were an estimated 10.6 million new cases of TB and 1.6 million TB deaths in 2021 [2]. The WHO has set ambitious targets to end the global TB epidemic by 2035, which include reducing TB deaths by 95%, TB incidence by 90%, and eliminating catastrophic costs for TB-affected households compared to 2015 levels [3].

However, achieving these targets faces many challenges, such as the emergence of drug-resistant TB, the co-infection of TB and HIV, a shortage of medical equipment, particularly in developing countries, and insufficient funding and political commitment for TB control [4–6]. Moreover, several social and environmental factors upsurge the risk of TB transmission and hinder access to and adherence to TB treatment. These factors include undernutrition, forced displacement, and homelessness, which often affect the most vulnerable and marginalized populations in the world [7, 8].

Undernutrition is a condition in which the body does not get enough nutrients to function properly. It can weaken the immune system and make people more susceptible to infections, such as TB. Undernutrition can also affect the response to TB treatment and increase the risk of mortality and relapse. According to the WHO, undernutrition is associated with an estimated 20% of all TB cases globally. Poverty, food insecurity, poor sanitation, and inadequate health care are common determinants of undernutrition.

Forced displacement exposes people to overcrowded and unsanitary living conditions, where TB can easily spread. Forced displacement can also disrupt the continuity of TB care and increase the loss of follow-up for TB patients [9]. According to the United Nations High Commissioner for Refugees (UNHCR), in 2022, the global burden of the forced displacement crisis had reached 108.4 million people, indicating a significant increase over time. This marked a rise of 19 million individuals compared to the end of 2021 [10]. Additionally, it is noteworthy that 70% of the world’s refugee population found refuge in low- and middle-income countries. Avoidable factors such as political instability, ethnic tensions, and human rights violations frequently precipitate forced displacement [11].

Homelessness is the lack of a fixed, regular, and adequate place to live [12]. It can expose people to harsh weather, violence, and social isolation, which can affect their physical and mental health. Homelessness can also limit access to health care services and social support, which are essential for the diagnosis and treatment of TB [13, 14]. Homelessness also plays a significant role in the burden of TB. Individuals experiencing homelessness often face multiple barriers to accessing healthcare services, including TB diagnosis and treatment [14]. Limited access to proper nutrition, inadequate shelter, and precarious living conditions further increase their vulnerability to TB infection and hinder treatment adherence [15].

Undernutrition, forced displacement, and homelessness are widely acknowledged as significant contributors to the incidence of tuberculosis (TB) and poor treatment outcomes. Despite recognition of their significance, gaps remain in understanding the complex interactions between these factors and TB on a global scale. Existing studies often focus narrowly on individual risk factors or specific populations, neglecting comprehensive trend analyses and long-term implications for achieving the World Health Organization’s (WHO) End TB targets. Hence, this study aims to bridge these gaps by analyzing trends from 2015 to 2022, elucidating the evolving patterns of these determinants and their impact on the TB incidence rate, as well as the feasibility and implications of achieving the End TB targets by 2035.

The selection of the data collection period from 2015 to 2022 corresponds with the adoption of the WHO’s End TB Strategy by the World Health Assembly in May 2014. This strategic alignment ensures that our analysis captures the implementation phase of the End TB Strategy, enabling us to assess progress towards its ambitious goals. By incorporating data spanning this timeframe, we aim to contribute to the monitoring and evaluation of global efforts to combat tuberculosis and inform evidence-based strategies for accelerating progress towards achieving the WHO’s targets for TB control and elimination by 2035.

## Methods

This study used a retrospective design to analyze the trends of undernutrition, conflict-related displacement, and homelessness from 2015 to 2022 and their effects on TB control efforts. The authors searched international databases such as UNHCR, Food and Agriculture Organization (FAO), and WHO for relevant and reliable data on these variables. They collected the data systematically from the selected sources and organized them chronologically to identify temporal trends and changes over time. They entered the data into Excel in chronological order to check for the availability of trend-related information. They used descriptive statistical analysis and trend analysis to measure the magnitude of the identified trends.

### Trend identification and interpretation

In our analysis, we utilized Microsoft Excel functions to identify and interpret trends within our dataset. Initially, we plotted the data points on a graph for visual trend assessment. To enhance our graphical representation, we added a trend line, offering a visual summary of data movements over time.

The trend line equation served as the basis for interpreting the trend analysis of our results. This equation provides a mathematical representation of the trend line, which enables us to forecast future values or examine the correlation between variables.

To ensure the reliability of our trend analysis, model fitness was checked using the coefficient of determination (R²). This statistical measure serves as a reliable indicator of how well the trend line aligns with the actual data points. R² value closer to 1 suggests a strong fit, meaning that our model accurately represents the data.

## Result

### Trend of undernutrition from 2015 to 2022

**Fig. 1:**
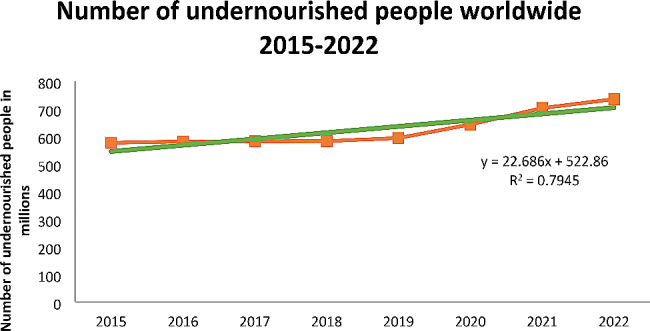
The trend of the Number of undernourished people worldwide from 2025 to 2022: Source UN Food and Agriculture Organization

Our trend analysis indicates a concerning escalation in the global prevalence of undernourishment from 2015 to 2022. Utilizing a trend line for data examination revealed a significant correlation, as evidenced by the equation y = 22.686x + 522.86 with an R² value of 0.7945 (see details in Fig. 1). Here, y represents the projected count of undernourished individuals in millions, x corresponds to the year, and the R² value confirms the trend line’s reliability in mirroring the data.

As presented in Fig. 1, the trend line reveals that each year, globally, 22.7 million people suffer from undernourishment. The data shows an alarming rise from 523 million in 2015 to 735 million by 2022. This trend underscores the escalating challenge of undernutrition and underscores the imperative for ongoing surveillance and strategic interventions to mitigate this pressing public health concern.

**Fig. 2:**
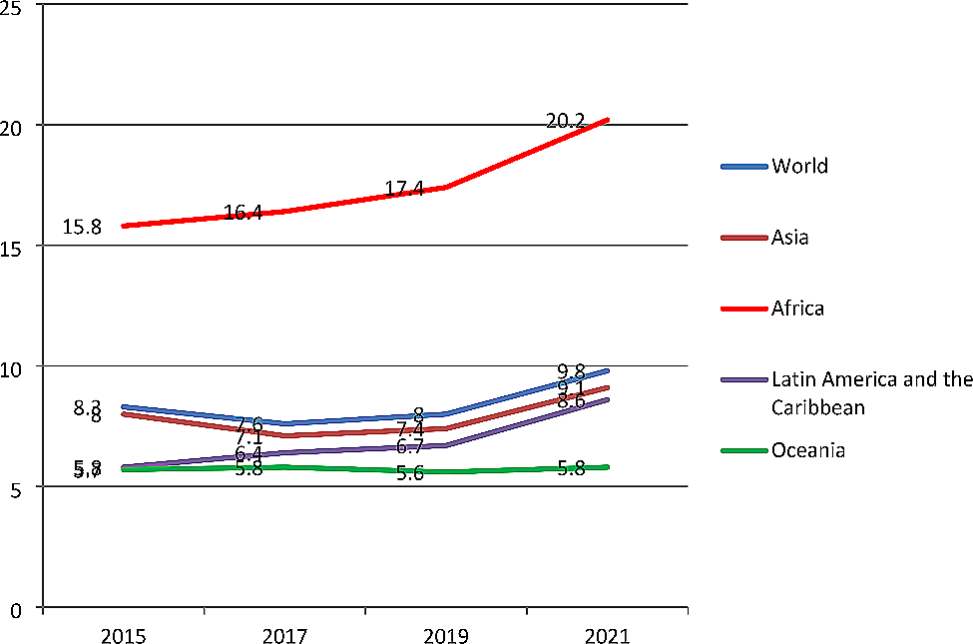
Prevalence of undernourished people from 2015 to 2021 and regional variation: Source UN Food and Agriculture Organization

When we see regional variation (as shown in Fig. 2), both Africa and Asia have the highest rates of undernutrition, and at the same time, they are huge TB-burden countries, while Oceania, Latin America, and the Caribbean have the lowest. This rise is concerning, particularly when compared to the 7.6% affected in 2017.

These stark realities exacerbate the already daunting challenge of achieving the World Health Organization’s End of TB Targets by 2035. The intricate connection between undernutrition and tuberculosis presents a significant impediment to TB eradication, necessitating comprehensive endeavors to address undernutrition, enhance food security, and concurrently prioritize initiatives for tuberculosis prevention and treatment.

### Trend of forced displacement from 2015 to 2022

**Fig. 3:**
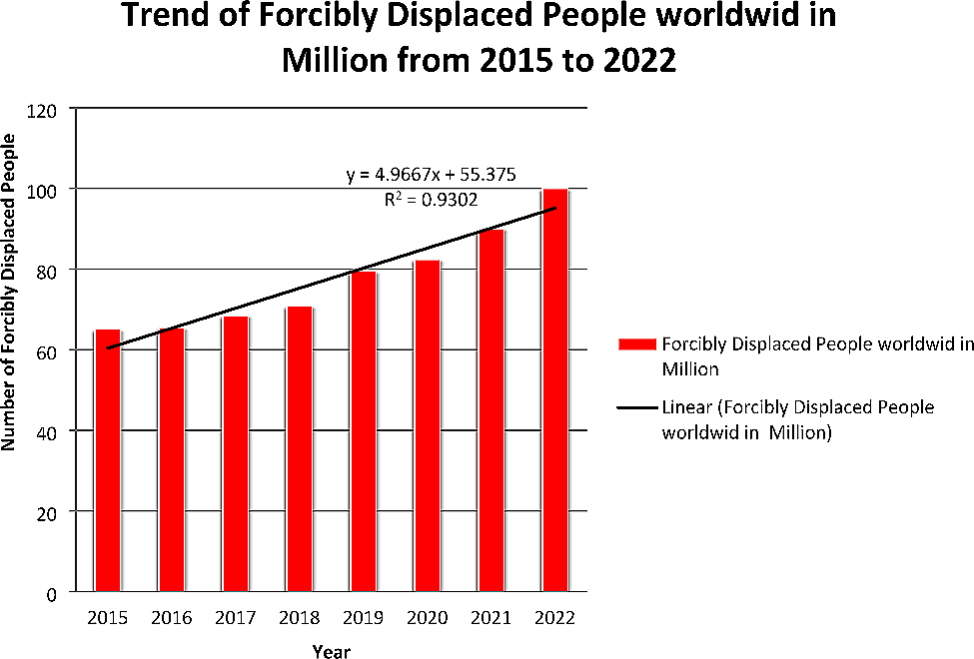
Trend of Forcibly Displaced People Worldwide in Million from 2015 to 2022: Source UNCHR

From 2015 to 2022, our analysis reveals a consistent annual increase in the number of forcibly displaced people worldwide. As illustrated in Fig. 3, the data proves that the number of individuals forcibly displaced due to conflict, persecution, violence, or human rights violations increased by approximately 5 million each year, culminating in a staggering total of 102.12 million in 2022. This signifies a remarkable surge of 52% from the 67.34 million recorded in 2015. To further examine this trend, our analysis involved fitting a linear model to the data, yielding the equation Y = 4.966x + 55.375, where Y represents the number of forcibly displaced people worldwide in millions and x denotes the year (see details in Fig. 3). Notably, our model exhibits a high coefficient of determination (R2 = 0.93), indicating a strong linear relationship between the year and the number of forcibly displaced people. These findings underscore the alarming magnitude and persistence of the global displacement crisis, which is profoundly impacting millions of individuals globally.

### Trend of homelessness

Globally, approximately 1.6 billion people live in inadequate housing conditions, and the issue of homelessness has seen a concerning increase over the past decade, with around 15 million individuals being forcefully evicted each year. Concurrently, homelessness is often overlooked in the planning and implementation of TB control programs, despite the World Health Organization’s Global Tuberculosis Report 2022 indicating that TB rates are significantly higher among people experiencing homelessness. This neglect is due to factors such as overcrowded living conditions, poor nutrition, and limited healthcare access, which contribute to the exacerbation of TB spread. Our study reinforces that homeless individuals are a highly vulnerable group to TB, yet they remain marginalized in effective intervention strategies for TB control, highlighting a critical gap in public health efforts to address both homelessness and TB globally.

Despite the fact that homelessness is a highly vulnerable group to TB, accurate homelessness figures are not yet available from an international database. The lack of accurate data from international databases significantly hinders and complicates the WHO’s intervention strategies to end TB target by 2035 and highlights how much homelessness is overlooked in TB control efforts. Due to the absence of numerical data, graphical representations of the trend of homelessness are not available.

### TB incidence rate

Globally, the estimated TB incidence rate (new cases per 100,000 population per year) was 133 (95% UI: 124–143) in 2022. The net reduction from 2015 to 2022 was 8.7%, far from the WHO End TB Strategy milestone of a 50% reduction by 2025. Additionally, the global targets set at the first UN high-level meeting on TB for the 5 years from 2018 to 2022 were not met, falling 50% short of the target despite the time period ending.

**Fig. 4:**
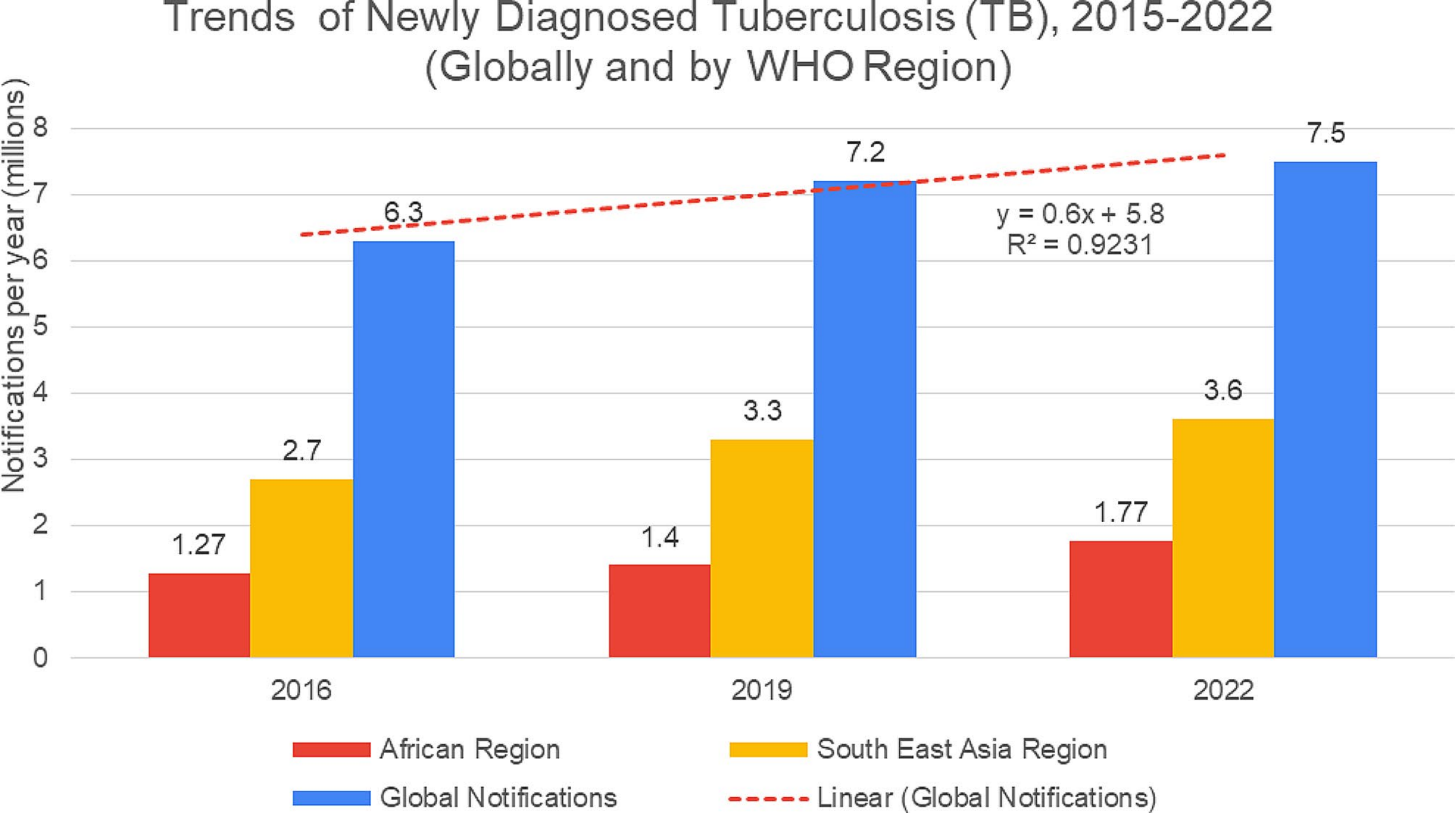
Trends of Newly Diagnosed Tuberculosis (TB), 2015–2022 (Globally and by WHO Region)

As a comparison, we can check (in Fig. 4, adopted from the WHO report on tuberculosis 2023) show that, the trend of newly diagnosed cases of tuberculosis increased over time, from 2015 to 2022, globally, in Africa, and Southeast Asia. This trend is directly proportional to undernutrition, forced displacement and homelessness. The increased trend graph and the R square value justify it as a true trend.

## Discussion

Our trend analysis has shown that hunger and undernutrition have increased globally from 2015 to 2022 over time, which has significant implications for the incidence of tuberculosis (TB) and the achievement of the World Health Organization’s (WHO) End TB targets. According to the WHO, undernutrition is identified as one of the most significant drivers of TB, accounting for an estimated 19% of incident TB worldwide in 2020 [15]. Studies reveal that People with undernutrition are three times more likely to develop TB disease [16, 17]. Undernutrition impairs the immune response and the vaccine response to TB, which can lead to an increased risk of infection, progression, and transmission [18]. Moreover, undernutrition can worsen the severity of TB and lead to poorer treatment outcomes, including higher rates of death and relapse [19, 20].

Our trend analysis shows that forced displacement is on the rise over time. Addressing this issue necessitates a comprehensive and coordinated effort from various stakeholders, including the government, health authorities, humanitarian agencies, donors, and civil society, to achieve the End TB targets. Forced displacement can increase the risk of TB transmission among displaced populations, as they often live in overcrowded, unsanitary, and insecure conditions [21, 22]. This can also facilitate the spread of drug-resistant TB strains, which are harder and more expensive to treat [23].

According to World Health Organization (WHO) data, studies conducted among internally displaced persons (IDPs) in North-East Nigeria and Sudan, areas deeply affected by political conflict and insurgency, have identified a negative impact on TB case detection and treatment outcomes, especially among the displaced population. The research findings pinpointed key factors influencing the access and utilization of TB services among IDPs, including security concerns, social stigma, and a lack of awareness [24]. Forced displacement can disrupt the continuity of TB services, such as diagnosis, treatment, and follow-up [25]. Displaced people may face barriers to accessing health facilities, such as distance, cost, security, or stigma. They may also lose their medical records, medications, or contact with health workers during the displacement [26, 27]. This can lead to delays in diagnosis, treatment interruptions, poor outcomes, and increased mortality [22].

So, forced displacement can hamper the implementation of the End TB Strategy, which requires a multi-sectoral and people-centered approach to end the TB epidemic. Conflict and displacement can undermine the political commitment, financial resources, and coordination mechanisms needed to achieve the strategy’s goals and targets.

Our review underscores the increasing global burden of homelessness over time, posing a significant challenge to ending the end-TB target by 2035. Homelessness serves as a major risk factor for TB, exposing individuals to TB bacteria in crowded and unsanitary environments [28, 29]. Immune stressors associated with homelessness, like rough sleeping, poor nutrition, and substance abuse, heighten the likelihood of TB development [30, 31]. Homeless individuals often delay seeking medical help due to misinterpreting TB symptoms and face barriers to accessing and adhering to TB treatment, including a lack of social support and stable housing [32, 33].

Studies from 1994 to 20,104 in the United States show that the incidence of TB was more than 10 times higher among homeless individuals compared to people not experiencing homelessness [34]. In Ethiopia, homeless individuals have approximately 4.67 times higher burdens of smear-positive PTB than the general population [35]. Additionally, the incidence of TB among homeless individuals is four times higher than that in the general population [36]. Furthermore, the diagnosis, treatment, and prevention of TB among homeless individuals are challenging [37]. Some of the barriers to TB control among homeless individuals include a lack of awareness, stigma, distrust, mobility, and competing priorities [38, 39].

According to the WHO Tuberculosis Report 2022, the COVID-19 pandemic has had a profound impact on global health systems, disrupting essential services and likely exacerbating challenges in meeting the WHO’s goals to end TB. Although quantifying these impacts is complex, it is recognized that the pandemic has likely worsened overall numbers, as evidenced by the significant decline in TB case notifications and an estimated increase in tuberculosis deaths in the peak years of 2020 and 2021.

## Recommendations for policymakers and program managers

To achieve end-TB targets, the WHO and the CDC advocate for primary prevention over secondary and tertiary prevention. However, TB remains a persistent threat, especially for vulnerable and marginalized groups. Based on our findings, we propose the following recommendations to achieve progress toward the end-TB targets set by the WHO:

1. Develop and implement targeted nutrition interventions to address the increasing rates of undernutrition, particularly in regions with the highest prevalence, such as Africa and Asia. This may include expanding access to nutritious food, micronutrient supplementation programs, and nutrition education initiatives aimed at vulnerable populations.
2. Allocate resources and implement tailored interventions to support forcibly displaced populations, including refugees, IDPs, and asylum seekers. This could involve strengthening social protection systems, providing access to healthcare services, and facilitating access to safe and stable housing options to mitigate the adverse effects of displacement on TB transmission and control.
3. Implement comprehensive homelessness prevention and support services to address the worsening rates of homelessness globally. This may involve increasing access to affordable housing options, providing supportive services such as mental health and substance abuse treatment, and implementing strategies to prevent homelessness among at-risk populations.

By implementing these targeted actions, policymakers and program managers can address the specific challenges identified in the trend analysis and advance progress toward achieving the End TB Targets by 2035.

### Strengths and limitations

Strengths of the study include its relevance and timeliness in addressing global health concerns, particularly the ambitious goal of achieving the End TB Targets set by the WHO, alongside its comprehensive analysis integrating socioeconomic factors like undernutrition, forced displacement, and homelessness.

This trend analysis offers valuable insights into the trajectory of TB incidence and its relationship with underlying social determinants over time. Furthermore, the findings have significant policy implications, emphasizing the importance of addressing these factors in TB control strategies to inform more effective and inclusive policies aimed at achieving the End TB Targets. However, the study is limited by establishing causality versus correlation between TB and socioeconomic factors and potential issues regarding generalizability and projection assumptions. Additionally, the complexity of interactions among various determinants may not be fully captured within the study’s scope, highlighting the need for further research in this area.

Furthermore, our study is the lack of specific analysis regarding the direct impact of the COVID-19 pandemic on the observed trends. While we acknowledge the potential influence of global disruptions, such as supply chain interruptions and the strain on public health programs, including those addressing tuberculosis, undernutrition, and poverty, our study did not quantitatively assess these effects.

## Conclusion

We found that undernutrition, forced displacement, and homelessness have worsened significantly over time, which makes it very hard to meet the End-TB Targets by 2035 without addressing these interrelated root causes. These factors increase the vulnerability of the community to TB infection, transmission, and treatment failure, as well as reduce their access to quality health care and social protection. Therefore, we recommend that TB programs adopt a comprehensive and multi-sectoral approach that addresses the underlying determinants of TB burden and improves the health and well-being of affected populations.

## Data Availability

The datasets used and/or analysed during the current available from the corresponding author on reasonable request.

## Abbreviations

FAO: Food and Agriculture Organization
TB: Tuberculosis
UNHCR: United Nations High Commissioner for Refugees
WHO: World Health Organization
